# Astaxanthin as a Therapeutic Candidate for Nociceptive and Inflammatory Pain: Mechanisms and Perspectives

**DOI:** 10.3390/md24030101

**Published:** 2026-03-03

**Authors:** Mamoru Takeda, Risako Chida

**Affiliations:** Laboratory of Food and Physiological Sciences, Department of Life and Food Sciences, School of Life and Environmental Sciences, Azabu University, 1-17-71, Fuchinobe, Chuo-ku, Sagamihara 252-5201, Kanagawa, Japan; f22001@azabu-u.ac.jp

**Keywords:** astaxanthin, carotenoid, complementary alternative medicine, trigeminal nociceptive neuron, electrophysiology, nociception, inflammatory pain, hyperalgesia, pathological pain, celecoxib, non-steroidal anti-inflammatory drugs

## Abstract

Recently, complementary and alternative medicine (CAM) has been actively employed for patients experiencing symptoms unresponsive to Western medical treatments like drug therapy. The natural compounds carotenoids and astaxanthin (AST) have demonstrated various beneficial biological actions for human health in several studies. Given their broad pharmacological activities and reduced toxicity, ASTs possess significant potential as resources for the development of natural analgesic drugs. Given recent studies showing that AST can modulate neuronal excitability, including nociceptive sensory transmission through voltage-gated Ca^2+^ channels and the n-methyl-D-aspartate (NMDA) glutamate receptor, and inhibit the cyclooxygenase-2 cascade, AST holds promise as a CAM, particularly as a therapeutic agent for nociceptive and pathological pain. Based on the in vivo research findings from our laboratory presented in this review, we have confirmed that carotenoid ASTs possess: (i) an intravenous anesthetic effect on both nociceptive and inflammatory pain comparable to existing analgesics such as ketamine; and (ii) an anti-inflammatory effect on chronic pain with an efficacy almost equivalent to that of the commonly used non-steroidal anti-inflammatory drug (NSAID) celecoxib. Therefore, these findings suggest that, as natural compounds, ASTs contribute to the relief of nociceptive and inflammatory pain, implying their potential for clinical application.

## 1. Introduction

Conventional Western pharmacological treatments for chronic pain often present limitations for certain patient populations, leading to a rising clinical interest in Complementary and Alternative Medicine (CAM) [1,2,3]. Although CAM—specifically herbal remedies and acupuncture—is sometimes characterized in Western medicine as lacking exhaustive scientific validation, a growing body of evidence highlights the therapeutic potential of natural compounds. These substances have demonstrated substantial biological activities, including antioxidant, anti-inflammatory, and cardioprotective properties, in addition to their antinociceptive effects [4,5,6,7,8].

The clinical utility of traditional analgesics is frequently hindered by severe adverse reactions associated with long-term administration. Consequently, there is a critical need to identify safer natural alternatives that provide therapeutic efficacy with minimal toxicity. Given their diverse pharmacological profiles and favorable safety margins, natural compounds represent a promising foundation for novel analgesic development. While existing local anesthetics and non-steroidal anti-inflammatory drugs (NSAIDs) are effective, they are not without significant risks; for instance, inflammation can compromise the efficacy of local anesthetics in dental contexts [9]. Furthermore, while NSAIDs such as celecoxib effectively inhibit cyclooxygenase-2 (COX-2) and prostaglandin E_2_ (PGE_2_) synthesis, their use is clinically linked to gastrointestinal ulcers and increased cardiovascular risks, such as myocardial infarction [10].

In this context, phytochemicals and fatty acids have emerged as viable CAM strategies for managing pain within inflamed tissues [11]. These compounds, derived from various dietary sources including fruits, vegetables, and marine life, encompass diverse groups such as polyphenols and carotenoids. Among these, Astaxanthin (AST)—a xanthophyll carotenoid prevalent in microalgae, crustaceans, and salmonids—has garnered particular attention [12]. AST exhibits superior antioxidant capacity compared to other carotenoids like lutein and zeaxanthin [13,14,15], and possesses a broad biological spectrum including anti-tumor, anti-diabetic, and immunomodulatory activities [16,17,18].

While the prior literature has reviewed the antinociceptive potential of carotenoids in animal models [10,19,20], a detailed synthesis of their neurophysiological mechanisms remains limited. Specifically, there is a paucity of data regarding how carotenoids influence the excitability of nociceptive neurons under pathological conditions, particularly through in vivo electrophysiological assessments. Nevertheless, recent in vitro evidence suggests that AST modulates neuronal excitability by interacting with key molecular targets, such as voltage-gated Ca^2+^ (Cav) channels, N-methyl-D-aspartate (NMDA) receptors, and COX-2 [21,22,23,24,25]. Recent work by Zhao et al. [26] showed that AST exerts potent inhibitory effects on neuroinflammation and the activation of phosphorylation of extracellular signal-regulated kinase (p-ERK)1/2, phosphorylation of p38 mitogen-activated protein kinase (p38 MAPK), and nuclear factor-kB (NF-κB) p65 nuclear translocation. Consequently, AST holds promise as a new therapeutic tool for treating neuropathic pain. In addition, Zhao et al. [27] demonstrated that AST exhibits anti-inflammatory properties mediated by p38 MAPK and nuclear factor erythroid 2-related 2 (Nrf2) /heme oxygenase-1(HO-1) in inflammatory pain models, highlighting its potential utility as a pharmacological intervention for inflammatory pain relief.

Our laboratory has recently utilized neurophysiological methods to evaluate the analgesic properties of dietary AST. Our published findings demonstrate that both acute intravenous administration and chronic intake of AST significantly attenuate nociceptive and inflammatory pain [23,24,25]. The present review aims to synthesize current knowledge regarding the role of AST in relieving nociceptive and pathological pain, discussing its potential clinical utility and the significance of its chemical structure in mediating analgesic effects, with a particular focus on recent in vivo evidence [23,24,25]. Although this study focuses on the molecular mechanisms in animal models, these findings provide a necessary foundation for future clinical applications and the development of functional foods.

## 2. Clinical Manifestations of Nociceptive and Pathological Pain

The critical biological necessity of pain is perhaps best illustrated by the rare genetic condition known as congenital insensitivity to pain (congenital analgesia). Individuals with this disorder possess dysfunctional nociceptors [28], rendering them entirely unable to perceive painful stimuli. This sensory deficit leads to catastrophic clinical outcomes, including recurrent fractures, joint deformities, and life-threatening complications such as sepsis or tissue necrosis from undetected burns. These severe consequences underscore the role of pain as an indispensable “biological warning system” that alerts the organism to potential or actual tissue damage, thereby ensuring survival [29]. This protective mechanism is the primary function of physiological (nociceptive) pain.

In contrast, pathological pain represents a maladaptive state in which this “biological warning system” becomes dysfunctional. This condition is defined by neuroplastic alterations in pain-transmission neurons and the persistence of nociceptive signaling, which often continues long after the initial tissue injury has resolved [29,30]. Pathological pain is generally categorized into inflammatory and neuropathic pain. Inflammatory pain results from the peripheral sensitization of nociceptors by biochemical mediators, such as PGE_2_, at the site of injury or inflammation (e.g., thermal burns or rheumatoid joints) [31].

Neuropathic pain, on the other hand, stems from direct damage to the somatosensory nervous system and persists independently of the original wound healing. Common clinical manifestations include diabetic peripheral neuropathy, sciatica, and trigeminal neuralgia. In the context of dentistry, neuropathic pain is frequently encountered as a post-operative complication following invasive procedures such as tooth extractions or dental implantation.

## 3. Trigeminal Pain Transmission Pathway: Overview

**Figure 1:**
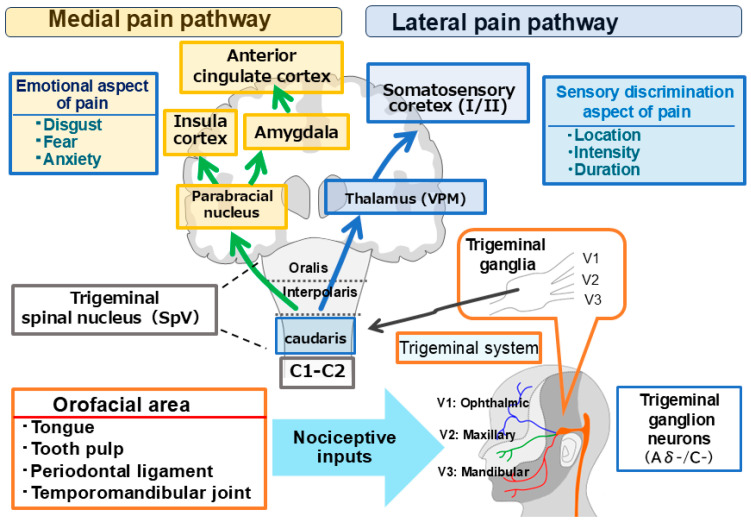
Ascending trigeminal pathways for nociceptive processing. Trigeminal pain signaling is organized into two parallel systems: the lateral pathway, which mediates sensory-discriminative functions, and the medial pathway, which processes affective-motivational dimensions. Noxious stimuli from orofacial tissues are transduced by Aδ- and C-fibers of the trigeminal ganglion, projecting to the spinal trigeminal nucleus caudalis (SpVc) and the upper cervical dorsal horn (C1–C2). From this complex, the lateral pathway ascends via the ventral posteromedial (VPM) thalamic nucleus to the primary and secondary somatosensory cortices (S1/S2). Simultaneously, the medial pathway relays information through the parabrachial nucleus to limbic structures, including the anterior cingulate and insular cortices.

This review focuses on the potential contributions of AST to the alleviation of nociceptive and pathological pain, necessitating an understanding of the general characteristics of trigeminal pain pathways and nociceptive neurons (Figure 1). In the trigeminal nervous system, pain signaling is functionally bifurcated into the lateral and medial systems: the former transmits sensory-discriminative details such as pain intensity and topographical location, while the latter conveys the affective-motivational components, including the emotional perception of “unpleasantness” [32,33,34]. Nociceptive inputs from orofacial regions—including the tongue, dental pulp, periodontal ligament, and temporomandibular joint—are initially carried by trigeminal ganglion (TG) neurons to the trigeminal spinal nucleus caudalis (SpVc) and the upper cervical dorsal horn (C1–C2) [32,35]. At this junction, two distinct neuronal classes process these signals: Wide Dynamic Range (WDR) neurons, which respond to both non-nociceptive and nociceptive stimuli by increasing discharge frequency in correlation with stimulus intensity, and nociceptive-specific (NS) neurons, which react exclusively to noxious stimuli to facilitate spatial localization. Notably, WDR neurons are implicated in the development of hyperalgesia following tissue injury and inflammation [32,35]. From the SpVc/C1-2 complex, sensory-discriminative information is relayed via the ventral posteromedial thalamic nucleus to the primary and secondary somatosensory cortices; simultaneously, affective data are transmitted through the parabrachial and medial thalamic nuclei to the amygdala, insular cortex, and anterior cingulate cortex for comprehensive emotional interpretation [32,35].

## 4. Mechanisms of Pain Transmission: Electrical and Chemical Signaling

**Figure 2:**
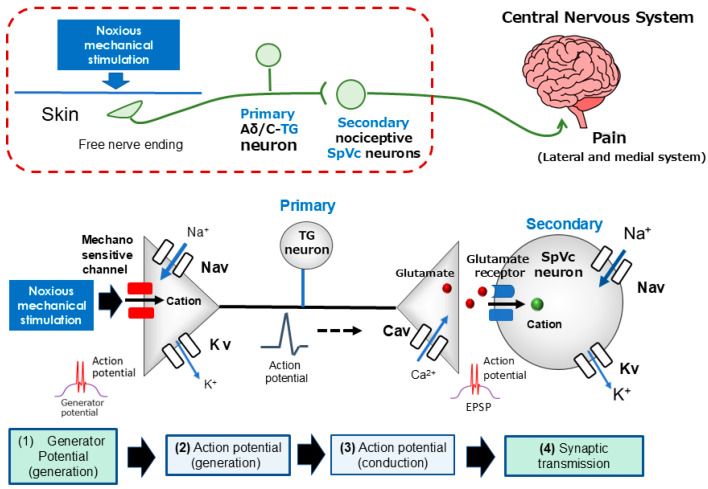
Signaling cascade of trigeminal nociceptive transduction and synaptic transmission. The process initiates when noxious mechanical stimuli trigger a depolarizing generator potential at the peripheral terminals of trigeminal ganglion (TG) neurons. This depolarization activates voltage-gated sodium (Nav) and potassium (Kv) channels, resulting in the generation and axonal conduction of action potentials toward the central terminals in the spinal trigeminal nucleus caudalis (SpVc). Upon reaching the presynaptic terminal, the arrival of action potentials induces the opening of voltage-gated calcium (Cav) channels, facilitating the exocytosis of neurotransmitters into the synaptic cleft. The subsequent binding of these neurotransmitters to postsynaptic ionotropic glutamate receptors generates excitatory postsynaptic potentials (EPSPs). When the EPSP amplitude exceeds the excitation threshold, a sequence of action potentials is propagated to higher encephalic centers, leading to the conscious perception of pain.

The transmission of nociceptive information relies on primary afferent fibers, specifically the pseudounipolar neurons of the trigeminal ganglion (TG), which are classified into thin, myelinated Aδ fibers and unmyelinated C fibers [32,35]. These fibers serve distinct functional roles: Aδ fibers mediate rapid, sharply localized “fast” pain, while C fibers conduct more diffuse, “slow” aching sensations [32,35]. Structurally, TG neurons extend peripheral axons that terminate as free nerve endings and central axons that establish chemical synapses with second-order neurons in the central nervous system (CNS). As illustrated in Figure 2, this sensory processing sequence encompasses four fundamental stages: (i) transduction of external noxious energy into electrical signals, (ii) generation of action potentials, (iii) conduction along the axon, and (iv) synaptic transmission within the CNS [35,36].

At the peripheral terminals, noxious mechanical, thermal, or chemical stimuli activate specialized mechanosensitive ion channels, such as TRPA1 and ASICs [37,38,39,40]. This activation enhances cation permeability, triggering an inward current that produces a depolarizing generator potential. As a non-propagating analog signal, the amplitude of this generator potential is proportional to the stimulus intensity; once it reaches a critical threshold, it facilitates the transition from an analog signal to “all-or-none” digital action potentials for long-distance propagation.

The initiation of pain signaling begins with the generation of a non-propagating receptor potential at the nociceptor terminal. Once this graded potential reaches a critical threshold in response to noxious stimuli, it triggers an action potential—a rapid electrochemical event characterized by a depolarization phase, mediated by Na^+^ influx through voltage-gated sodium (Nav) channels, followed by a repolarization phase driven by K^+^ efflux via voltage-gated potassium (Kv) channels [35,36]. Nociceptive neurons express a combination of tetrodotoxin-sensitive (TTX-S) and tetrodotoxin-resistant (TTX-R) Nav channels; while Aδ fibers utilize both subtypes, C fibers predominantly express the TTX-R variant [41]. Crucially, the amplitude of the initial generator potential is proportional to the stimulus intensity, which in turn modulates the firing frequency of the subsequent action potentials. These signals propagate actively along the axon to the central terminals, where the arrival of the impulse activates voltage-gated calcium (Cav) channels. The resulting Ca^2+^ influx into the presynaptic terminal facilitates the exocytosis of excitatory neurotransmitters, primarily glutamate, into the synaptic cleft. These neurotransmitters bind to postsynaptic ionotropic glutamate receptors, generating an excitatory postsynaptic potential (EPSP). The amplitude of the EPSP, which is directly determined by the quantity of released neurotransmitter, dictates the firing frequency of the second-order neuron. This frequency-coded information is then transmitted to higher CNS centers for the comprehensive processing and encoding of pain intensity [32,35].

## 5. Modulation of Nociceptive Transmission by AST

The therapeutic potential of AST in modulating nociceptive transmission is supported by both its biochemical properties and recent electrophysiological evidence. Previous in vitro investigations have demonstrated that AST dose-dependently inhibits glutamate release from rat cortical synaptosomes by suppressing presynaptic N-type Cav channels and the mitogen-activated protein kinase (MAPK) signaling cascade [21]. Furthermore, AST has been shown to ameliorate neuropathic pain by antagonizing N-methyl-D-aspartate (NMDA) glutamate receptors [22]. Given its ability to cross the blood–brain barrier [42], systemic administration of AST likely modulates excitatory glutamatergic transmission within the central nervous system, including the trigeminal nociceptive pathways. Our recent findings further substantiate this hypothesis: (i) AST dose-dependently and reversibly inhibited the firing rates of SpVc WDR neurons in response to both noxious and non-noxious mechanical stimulation, whereas (ii) vehicle administration exerted no significant effect on spontaneous or evoked neuronal activity. Considering the established role of glutamate receptor antagonists in migraine management [43] and the NMDA-antagonistic effects of AST [22], it is plausible that AST may alleviate primary headache syndromes, such as migraine and cluster headache, potentially offering clinical efficacy comparable to the NMDA receptor blocker ketamine.

**Figure 3:**
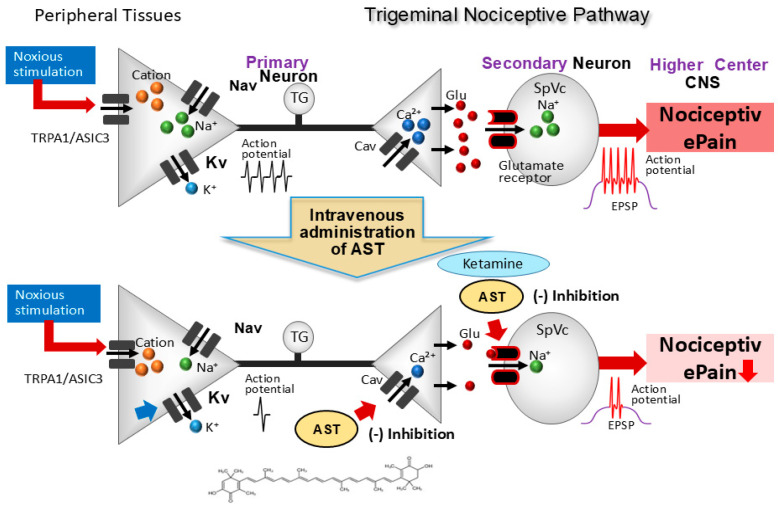
Proposed mechanisms of AST-mediated inhibition of SpVc WDR neuronal discharge. The nociceptive signaling cascade begins with noxious mechanical stimulation of the skin, which activates mechanosensitive ion channels (e.g., TRPA1, ASIC3), resulting in a depolarizing generator potential. This depolarization triggers the opening of Nav and Kv channels, generating action potentials that propagate along primary afferent fibers to the central terminals in the spinal trigeminal nucleus caudalis (SpVc). Upon reaching the presynaptic terminal, these action potentials induce the opening of Cav channels and subsequent Ca^2+^ influx. This rise in intracellular Ca^2+^ facilitates the exocytosis of excitatory neurotransmitters, primarily glutamate (Glu), into the synaptic cleft. The binding of glutamate to postsynaptic ionotropic receptors generates excitatory postsynaptic potentials (EPSPs); when these EPSPs reach the firing threshold, action potentials are initiated and transmitted to higher pain centers. Intravenous administration of AST is thought to suppress the excitability of SpVc WDR neurons by dual inhibition: suppressing presynaptic Cav channels to reduce neurotransmitter release and antagonizing postsynaptic glutamate receptors. This synergistic effect leads to a significant decrease in the firing frequency of SpVc WDR neurons, ultimately alleviating pain perception. TRPA1, transient receptor potential ankyrin 1; ASIC3, acid-sensing ion channel 3; EPSP, excitatory postsynaptic potential; Nav, voltage-gated sodium channel; Kv, voltage-gated potassium channel; Cav, voltage-gated calcium channel; Glu, glutamate.

Collectively, these results provide compelling evidence that acute intravenous AST suppresses trigeminal sensory transmission—even in the absence of pre-existing inflammation or neuropathy—likely through the inhibition of Cav channels and excitatory glutamatergic signaling (Figure 3). Consequently, AST represents a promising CAM candidate for the management of trigeminal nociceptive pain, potentially offering a favorable safety profile without the side effects associated with conventional NMDA antagonists such as ketamine.

## 6. Modulation of Pathological Pain by AST

### 6.1. AST as an Intravenous Therapeutic: Targeting Inflammatory Pain Pathways

The efficacy of AST in mitigating inflammatory pain is closely linked to its modulation of ion channel expression and synaptic hyperexcitability. Previous studies have reported a significant up-regulation of N-type Cav channels in dorsal root ganglion and spinal dorsal horn neurons under carrageenan-induced acute inflammation [44], as well as selective up-regulation of NMDA-NR1 receptors in colitis models [45]. These findings suggest that the sensitization of both presynaptic Cav channels and postsynaptic NMDA receptors is a primary driver of inflammatory hyperalgesia.

Recent investigations by Chida and Takeda [25] specifically addressed whether intravenous AST could alleviate the inflammatory hyperexcitability of SpVc WDR neurons. In a Complete Freund’s Adjuvant (CFA)-induced inflammatory model, which was characterized by significantly reduced mechanical escape thresholds and localized edema, AST demonstrated a dose-dependent and reversible inhibition of SpVc WDR neuronal firing. Notably, the maximal suppressive effect on noxious mechanical stimuli occurred within 15 min and persisted for 45 min. A key finding of this study was that AST preferentially inhibited neuronal responses to noxious stimulation over non-noxious inputs, an effect not observed in vehicle-treated controls. This preferential inhibition suggests that AST may specifically target the mechanisms underlying central sensitization, potentially through the dual action of suppressing inflammation-induced up-regulation of Cav and NMDA channels, and potentially enhancing descending inhibitory systems, such as endogenous opioidergic pathways. Collectively, these observations provide robust evidence that intravenous AST effectively suppresses noxious synaptic transmission within the SpVc during inflammation, positioning it as a promising therapeutic candidate for trigeminal inflammatory pain with a superior safety profile compared to conventional treatments.

### 6.2. AST for Chronic Inflammatory Pain Relief: Modulation of Pro-Inflammatory Cascades

The clinical potential of AST in alleviating chronic inflammatory pain is rooted in its ability to suppress key pro-inflammatory mediators. Studies have demonstrated that AST concentration-dependently inhibits the production of prostaglandin E2 (PGE_2_) and tumor necrosis factor-alpha (TNFα) in both in vivo and in vitro models [46]. Furthermore, AST suppresses the expression of cyclooxygenase-2 (COX-2), the enzyme responsible for PGE_2_ synthesis, in chondrocytes and microglial cells [47,48]. PGE2 is a critical driver of pain hypersensitivity; it sensitizes mechanosensitive channels (e.g., TRPA1 and ASIC) and modulates Nav and Kv channels through protein kinase A (PKA) activation via prostanoid E (EP) receptors at nociceptive terminals [49]. Additionally, PGE_2_ reduces the activity of glycinergic inhibitory interneurons within the spinal dorsal horn, further exacerbating the pain state [50].

**Figure 4:**
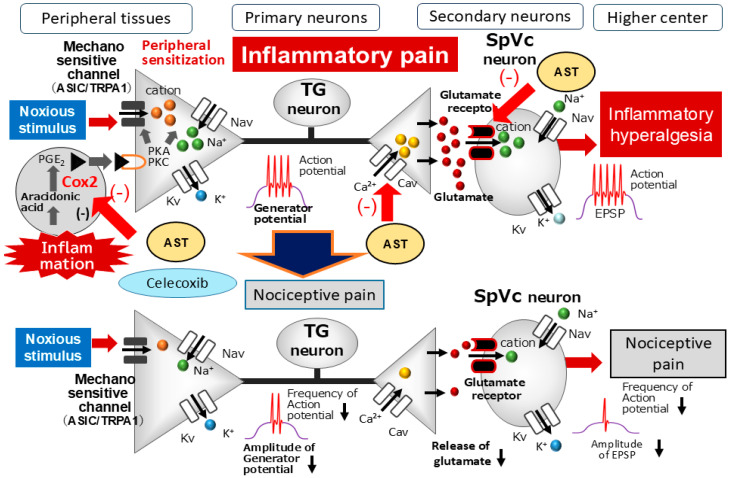
Proposed mechanisms by which Astaxanthin (AST) suppresses inflammation-induced mechanical hyperalgesia. Following peripheral tissue inflammation, inflammatory mediators such as PGE_2_ bind to G-protein-coupled prostanoid EP receptors. This interaction triggers the activation of protein kinase A (PKA) and protein kinase C (PKC) within nociceptive peripheral terminals, leading to the phosphorylation of mechanosensitive Na^+^ and K^+^ channels. Consequently, the activation thresholds for transducer channels, particularly the transient receptor potential (TRP) family (e.g., TRPA1), are significantly lowered. This reduction increases the membrane excitability of peripheral terminals, resulting in high-frequency action potential propagation to the central terminals of the spinal trigeminal nucleus caudalis (SpVc). This sustained input facilitates excessive glutamate release into the synaptic cleft, where it binds to up-regulated postsynaptic receptors, thereby augmenting excitatory postsynaptic potentials (EPSPs). The resulting barrage of action potentials conducted to higher encephalic centers manifests as a state of heightened sensitivity known as peripheral and central sensitization. Systemically administered AST is hypothesized to attenuate this hyperalgesia by suppressing the hyperexcitability of SpVc wide-dynamic range (WDR) neurons through a dual-action pathway: Peripheral Action: Inhibition of the cyclooxygenase (COX)-2 signaling cascade, which reduces the firing frequency of primary afferent nociceptors. Central Action: Attenuation of postsynaptic glutamate receptor activation and reduced Cav channel activity at the central terminals within the SpVc. Together, these synergistic mechanisms inhibit the conduction of noxious signals to higher brain regions, thereby modulating both the sensory-discriminative and affective-motivational dimensions of pain. Further studies are required to confirm the proposed potential mechanisms of AST in pain mitigation.

In our recent comparative study, the efficacy of systemic AST was evaluated against the selective COX-2 inhibitor celecoxib (CEL) in a CFA-induced orofacial inflammation model [24]. While inflamed rats exhibited marked mechanical hyperalgesia, treatment with AST, CEL, or a combined administration (1/2 CEL + 1/2 AST) successfully restored mechanical thresholds to baseline levels within two days. Electrophysiological recordings from SpVc WDR neurons further corroborated these behavioral results: all three treatment protocols significantly attenuated the heightened spontaneous activity, reduced the evoked firing rates in response to mechanical stimuli, and normalized the expanded receptive field sizes observed in inflamed subjects. These findings are consistent with prior evidence showing that other carotenoids, such as lutein, attenuate inflammatory hyperalgesia by suppressing COX-2 immunoreactivity in peripheral tissues [51]. Collectively, these observations suggest that AST mitigates mechanical hyperalgesia primarily by suppressing SpVc WDR neuronal hyperexcitability through the inhibition of the peripheral COX-2/ PGE_2_ cascade (Figure 4). When combined with its inhibitory effects on Cav channels and glutamate receptors, AST provides a multi-target approach to reducing nociceptive conduction, offering a robust foundation for developing novel analgesic strategies for orofacial pain with a potentially superior safety profile.

## 7. Functional Significance and Therapeutic Potential of AST

The increasing interest in CAM highlights the urgent need for effective alternatives when conventional Western pharmacotherapy proves inadequate. As detailed in this review, our in vivo neurophysiological research demonstrates that AST exerts potent analgesic and anti-inflammatory effects by targeting multiple molecular pathways. Specifically, AST exhibits: (i) an intravenous anesthetic-like effect on both nociceptive and inflammatory pain, comparable to established analgesics such as ketamine; and (ii) an anti-inflammatory efficacy equivalent to the selective COX-2 inhibitor celecoxib. These findings suggest that AST offers a promising clinical avenue for managing various pain states while potentially circumventing the adverse side effects associated with traditional drugs.

Furthermore, while this review primarily elucidated the sensory-discriminative aspects of pain (the lateral system), the potential impact of AST on the affective-motivational dimensions (the medial system) warrants further investigation. This is particularly relevant given the recent introduction of the term “nociplastic pain” by the International Association for the Study of Pain, describing pain arising from altered nociception despite no clear evidence of tissue or nerve damage [52,53]. Since nociplastic pain is heavily associated with central sensitization and emotional dysfunction, AST’s ability to reduce peripheral sensitization—a key driver of central hypersensitivity—positions it as a candidate for treating such complex conditions. Future research should prioritize the clinical translation of these findings, exploring AST’s role in drug discovery for functional foods and its broader applicability in alleviating the multifaceted pathology of nociplastic pain. Such advancements hold the potential to revolutionize pain management through the development of safer, non-pharmacological, or natural compound-based therapeutic modalities.

It should be noted that the previous studies cited in this review require further investigation into dose–response relationships and bioavailability. Additionally, given the current lack of human data, our conclusions remain speculative until further research provides more robust evidence.

## 8. Concluding Remark

Recent evidence highlighting the modulation of neuronal excitability—specifically the inhibition of Cav channels, glutamate receptors, and the COX-2 signaling cascade—positions AST as a compelling candidate for Complementary and Alternative Medicine (CAM). Our laboratory’s in vivo neurophysiological findings, as synthesized in this review, confirm that the carotenoid AST possesses a dual therapeutic profile: (i) an intravenous anesthetic-like effect on both nociceptive and inflammatory pain, with efficacy comparable to established analgesics such as ketamine; and (ii) a potent anti-inflammatory action against chronic pain, nearly equivalent to the selective COX-2 inhibitor celecoxib. Collectively, these data substantiate the role of natural compounds in the mitigation of acute and persistent pain, underscoring their significant potential for clinical integration as safe and effective analgesic agents. Future studies should focus on conducting robust clinical trials, refining administration protocols, and evaluating the optimal regulatory framework for AST, ranging from functional foods to pharmaceutical applications.”

## Data Availability

All data from this study are included in the main body of the article.

## Abbreviations

The following abbreviations are used in this manuscript:

AST	Astaxanthin
NSAID	Non-steroidal anti-inflammatory drug
CEL	Celecoxib
SpVc	Spinal Trigeminal nucleus caudalis
C1-C2	Upper cervical dorsal horn
WDR	Wide-dynamic range
NS	Nociceptive specific
TG	Trigeminal ganglion
CFA	Complete Freund’s Adjuvant
CAM	Complementary Alternative Medichine
COX-2	Cyclooxygenase-2
PGE_2_	Prostaglandine E2
MAPK	Mitogen activated protein kinase
NF-kB	Nuclear factor-kB
ERK	Extracellular signal regulated kinase
Nrf2	Nuclear erythroid-related2
HO-1	Heme oxygenase
PKA	Protein Kinase A
PKC	Protein Kinese C
NMDA	N-methyl-D-aspartate
NR2B	NMDA receptor subtype 2B
Nav	Voltage-gated Na channels
TTX-S	Tetrodotoxin-sensitive
TTX-R	Tetrodotoxin-resistant
Kv	Voltage-gated K channels
Cav	Voltage-gated Ca channels
TRPA1	Transient receptor protein ankyrn 1
ASIC3	Acid sensing ion channels 3
EPSP	Excitatory Post synaptic Potential
EP	Prostanoid E
TNFα	Tumor necrosis factorα
CNS	Central nervous syetem

## References

[1] Rao J.K., Mihaliak K., Kroenke K., Bradley J., Tierney W.M., Weinberger M. (1999). Use of complementary therapies for arthritis among patients of rheumatologists. Ann. Intern. Med..

[2] Konvicka J.J., Meyer T.A., McDavid A.J., Roberson C.R. (2008). Complementary/alternative medicine use among chronic pain clinic patients. J. Perianesth. Nurs..

[3] Rosenberg E.I., Genao I., Chen I., Mechaber A.J., Wood J.A., Faselis C.J., Kurz J., Menon M., O’Rorke J., Panda M. (2008). Complementary and alternative medicine use by primary care patients with chronic pain. Pain Med..

[4] Ernst E. (2003). Complementary medicine. Curr. Opin. Rheumatol..

[5] Fremont L. (2000). Biological effects of resveratrol. Life Sci..

[6] Pervaiz S. (2003). Resveratrol: From grapevines to mammalian biology. FASEB J..

[7] Shir Y., Raja S.N., Weissman C.S., Campbell J.N., Seltzer Z. (2001). Consumption of soy diet before nerve injury preempts the development of neuropathic pain in rats. Anesthesiology.

[8] Tall J.M., Raja S.N. (2004). Dietary constituents as novel therapeutics for pain. Clin. J. Pain.

[9] Miller R.D., Katzung B.G. (1996). Local Anesthetics. Basic and Clinical Pharmacology.

[10] Brune K., Zeilhofer H., McMahon S.B., Koltzenburg M. (2006). Antipyretic Analgesics: Basic Aspects. Wall and Melzack’s Textbook of Pain.

[11] Takeda M., Sashide Y. (2025). Pain managements with natural products: Neurophysiological insights. Int. J. Mol. Sci..

[12] Kuedo Z., Sangsuriyawong A., Klaypradit W., Tipmanee V., Chonpathompikunlert P. (2016). Effects of astaxanthin from *Litopenaeus vannamei* on carrageenan-induced edema and pain behavior in mice. Molecules.

[13] Hussein G., Sankawa U., Goto H., Matsumoto K., Watanabe H. (2006). Astaxanthin, a carotenoid with potential in human health and nutrition. J. Nat. Prod..

[14] Naguib T.M. (2000). Antioxidant activities of astaxanthin and related carotenoids. J. Agric. Food Chem..

[15] Liu X., Osawa T. (2009). Astaxanthin protects neuronal cells against oxidative damage and is a potent candidate for brain food. Forum Nutr..

[16] Jyonouchi H., Sun S., Iijima K., Gross M.D. (2000). Antitumor activity of astaxanthin and its mode of action. Nutr. Cancer.

[17] Guerin M., Huntley M.E., Olaizola M. (2003). *Haematococcus* astaxanthin: Applications for human health and nutrition. Trends Biotechnol..

[18] Chan K.C., Mong M.C., Yin M.C. (2009). Antioxidative and anti-inflammatory neuroprotective effects of astaxanthin and canthaxanthin in nerve growth factor differentiated PC12 cells. J. Food Sci..

[19] Boadas-Vaello P., Vala J.M., Verdu E. (2017). New pharmacological approaches using polyphenols on the physiology of neuropathic pain. Curr. Drug Targets.

[20] Tsuchiya H. (2017). Anesthetic agents of plant origin: A review of phytochemicals with anesthetic activity. Molecules.

[21] Lin T.Y., Lu C.W., Wang S.J. (2010). Astaxanthin inhibits glutamate release in rat cerebral cortex nerve terminals via suppression of voltage-dependent Ca^2+^ entry and mitogen-activated protein kinase signaling pathway. J. Agric. Food Chem..

[22] Sharma K., Sharma D., Sharma M., Sharma N., Bidve P., Prajapati P., Kalia K., Tiwari V. (2018). Astaxanthin ameliorates behavioral and biochemical alterations in in-vitro and in-vivo model of neuropathic pain. Neurosci. Lett..

[23] Chida R., Yamaguchi S., Utugi S., Sashide Y., Takeda M. (2024). Suppression of the excitability of nociceptive secondary sensory neurons following systemic administration of astaxanthin in rats. Anesth. Res..

[24] Chida R., Takeda M. (2025). Astaxanthin alleviates inflammatory mechanical hyperalgesia by reducing hyperexcitability of trigeminal nociceptive secondary neurons: Potential as an NSAID alternative. Molecules.

[25] Chida R., Takeda M. (2026). Acute intravenous astaxanthin administration modulates hyperexcitability in rat nociceptive secondary sensory neurons induced by inflammation. Mar. Drugs.

[26] Zhao L., Tao X., Song T. (2021). Astaxanthin alleviates neuropathic pain by inhibiting the MAPKs and NF-κB pathways. Eur. J. Pharmacol..

[27] Zhao L., Tao X., Wan C., Dong D., Wang C., Xi Q., Liu Y., Song T. (2021). Astaxanthin alleviates inflammatory pain by regulating the p38 mitogen-activated protein kinase and nuclear factor-erythroid factor 2-related factor/heme oxygenase-1 pathways in mice. Food Funct..

[28] Drissi I., Woods W.A., Woods C.G. (2020). Understanding the genetic basis of congenital insensitivity to pain. Br. Med. Bull..

[29] Cao H., Zhang Y.-Q. (2008). Spinal glial activation contributes to pathological pain states. Neurosci. Biobehav. Rev..

[30] Cervero F., Basbaum A.I., Bushnell M.C. (2013). Pain Theories. Science of Pain.

[31] Scholz J., Woolf C.J. (2002). Can we conquer pain?. Nat. Neurosci..

[32] Iwata K., Takeda M., Oh S.B., Shinoda M., Farah C.S., Balasubramaniam R., McCullough M.J. (2017). Neurophysiology of Orofacial Pain. Contemporary Oral Medicine.

[33] Sessle B.J. (2000). Acute and chronic craniofacial pain: Brainstem mechanisms of nociceptive transmission and neuroplasticity, and their clinical correlates. Crit. Rev. Oral Biol. Med..

[34] Shinoda M., Suzuro H., Iwata K., Hayashi Y. (2022). Plastic changes in nociceptive pathways contributing to persistent orofacial pain. J. Oral Biosci..

[35] Takeda M., Matsumoto S., Sessle B.J., Shinoda M., Iwata K. (2011). Peripheral and central mechanisms of trigeminal neuropathic and inflammatory pain. J. Oral Biosci..

[36] Harriott A.M., Gold M.S. (2009). Contribution of primary afferent channels to neuropathic pain. Curr. Pain Headache Rep..

[37] Price M.P., McIlwrath S.L., Xie J., Cheng C., Qiao J., Tarr T.E., Sluka K.A., Brennan T.J., Lewin G.R., Welsh M.J. (2001). The DRASIC cation channel contributes to the detection of cutaneous touch and acid stimuli in mice. Neuron.

[38] Kwan K.Y., Glazer J.M., Corey D.P., Rice F.L., Stucky C.L. (2009). TRPA1 modulates mechanotransduction in cutaneous sensory neurons. J. Neurosci..

[39] Borzan J., Zhao C., Mayer R.A., Raja S.N. (2010). A role for acid-sensing ion channel 3, but not acid-sensing ion channels 2, in sensing dynamic mechanical stimuli. Anesthesiology.

[40] Kang S., Jang J.H., Price M.P., Gautam M., Benson C.J., Geong H.G., Welsh M.J., Brennan T.J. (2012). Simultaneous disruption of mouse ASIC1a, ASIC2 and ASIC3 genes enhances cutaneous mechanosensitivity. PLoS ONE.

[41] Akopian A.N., Sivilotti L., Wood J.N. (1996). A tetrodotoxin-resistant voltage-gated sodium channel expressed by sensory neurons. Nature.

[42] Manabe Y., Komatsu T., Seki S., Sugawara T. (2018). Dietary astaxanthin can accumulate in the brain of rats. Biosci. Biotechnol. Biochem..

[43] Chan K., MaassenVanDenBrink A. (2014). Glutamate receptor antagonists in the management of migraine. Drugs.

[44] Yokoyama K., Kurihara T., Makita K., Tanabe T. (2003). Plastic change of N-type Ca channel expression after preconditioning is responsible for prostaglandin E2-induced long-lasting allodynia. Anesthesiology.

[45] Zhou Q.Q., Caudle R.M., Price D.D., Valle-Pinero A.Y.D., Verne G.N. (2006). Selective up-regulation of NMDA-NR1 receptor expression in myenteric plexus after TNBS induced colitis in rats. Mol. Pain.

[46] Ohgami K., Shiratori K., Kotake S., Nishida T., Mizuki N., Yazawa K., Ohno S. (2003). Effect of astaxanthin on lipopolysaccharide-induced inflammation in vitro and in vivo. Investig. Ophthalmol. Vis. Sci..

[47] Choi S.K., Park Y.S., Choi D.K., Chang H.I. (2008). Effects of astaxanthin on the production of NO and the expression of COX-2 and iNOS in LPS-stimulated BV2 microglial cells. J. Microbiol. Biotechnol..

[48] Peng J.J., Lu J.W., Liu F.C., Lee C.H., Lee H.S., Ho Y.J., Hsieh T.H., Wu C.C., Wang C.C. (2020). Astaxanthin attenuates joint inflammation induced by monosodium urate crystals. FASEB J..

[49] Takeda M., Takehana S., Sekiguchi K., Kubota Y., Shimazu Y. (2016). Modulatory mechanism of nociceptive neuronal activity by dietary constituents resveratrol. Int. J. Mol. Sci..

[50] Ahmadi S., Lippross S., Neuhuber W.L., Zeilhofer H.U. (2002). PGE2 selectively blocks inhibitory glycinergic neurotransmission onto rat superficial dorsal horn neurons. Nat. Neurosci..

[51] Syoji Y., Kobayashi R., Miyamura N., Hirohara T., Kubota Y., Uotsu N., Yui K., Shimazu Y., Takeda M. (2018). Suppression of hyperexcitability of trigeminal nociceptive neurons associated with inflammatory hyperalgesia following systemic administration of lutein via inhibition of cyclooxygenase-2 cascade signaling. J. Inflamm..

[52] Fitzcharles M.A., Cohen S.P., Clauw D.J., Littlejohn G., Usui C., Häuser W. (2021). Nociplastic pain: Towards an understanding of prevalent pain conditions. Lancet.

[53] Yoo Y.M., Kim K.H. (2024). Current understanding of nociplastic pain. Korean J. Pain.

